# Impact of Silicon Carbide Coating and Nanotube Diameter on the Antibacterial Properties of Nanostructured Titanium Surfaces

**DOI:** 10.3390/ma17153843

**Published:** 2024-08-02

**Authors:** Patricia dos Santos Calderon, Aravindraja Chairmandurai, Xinyi Xia, Fernanda G. Rocha, Samira Esteves Afonso Camargo, Kesavalu Lakshmyya, Fan Ren, Josephine F. Esquivel-Upshaw

**Affiliations:** 1 Department of Dentistry, Federal University of Rio Grande do Norte, Natal 59056, RN, Brazil; patriciascalderon@yahoo.com.br; 2 Department of Periodontology, College of Dentistry, University of Florida, Gainesville, FL 32610, USA; 3 Department of Chemical Engineering, University of Florida, Gainesville, FL 32611, USA; 4 Department of Oral Biology, College of Dentistry, University of Florida, Gainesville, FL 32610, USA; 5 Department of Comprehensive Oral Healthy, Adams Dental School, University of North Carolina, Chapel Hill, NC 27599, USA; 6 Department of Restorative Dental Sciences, Division of Prosthodontics, College of Dentistry, University of Florida, Gainesville, FL 32610, USA

**Keywords:** nanotechnology, surface engineering, biomedical materials, bacterial adhesion

## Abstract

This study aimed to comprehensively assess the influence of the nanotube diameter and the presence of a silicon carbide (SiC) coating on microbial proliferation on nanostructured titanium surfaces. An experiment used 72 anodized titanium sheets with varying nanotube diameters of 50 and 100 nm. These sheets were divided into four groups: non-coated 50 nm titanium nanotubes, SiC-coated 50 nm titanium nanotubes, non-coated 100 nm titanium nanotubes, and SiC-coated 100 nm titanium nanotubes, totaling 36 samples per group. *P. gingivalis* and *T. denticola* reference strains were used to evaluate microbial proliferation. Samples were assessed over 3 and 7 days using fluorescence microscopy with a live/dead viability kit and scanning electron microscopy (SEM). At the 3-day time point, fluorescence and SEM images revealed a lower density of microorganisms in the 50 nm samples than in the 100 nm samples. However, there was a consistently low density of *T. denticola* across all the groups. Fluorescence images indicated that most bacteria were viable at this time. By the 7th day, there was a decrease in the microorganism density, except for *T. denticola* in the non-coated samples. Additionally, more dead bacteria were detected at this later time point. These findings suggest that the titanium nanotube diameter and the presence of the SiC coating influenced bacterial proliferation. The results hinted at a potential antibacterial effect on the 50 nm diameter and the coated surfaces. These insights contribute valuable knowledge to dental implantology, paving the way for developing innovative strategies to enhance the antimicrobial properties of dental implant materials and mitigate peri-implant infections.

## 1. Introduction

Titanium and its alloys stand out as the predominant materials for dental implants, owing to their favorable physical attributes and biocompatibility. However, the risk of peri-implant infection remains a significant factor contributing to implant failure. Several factors can lead to infections, including inaccuracies during surgery, prolonged surgical procedures, and post-surgery contamination from adjacent tissues [1]. Dental implants are vulnerable to mucositis and peri-implantitis, which affect the supportive tissues. Peri-implantitis, characterized by irreversible bone loss around the implant, has an average prevalence of 9% in patients participating regularly in prophylaxis programs and 19% in those without regular maintenance [2].

Titanium (Ti) corrosion has been shown to initiate inflammatory reactions and could contribute to the progression of peri-implantitis. The pathogenesis of this disease is theorized to be an initiating reaction that leads to a shift in increased pathogenic bacteria, which in turn leads to the activation of an inflammatory mechanism. This mechanism cascades into a vicious cycle of bone loss, titanium corrosion from the decreased environmental pH, and the progression of inflammation.

Pathogens commonly associated with periodontal diseases could be significant in peri-implantitis [3,4]. Shibli et al. [3] found significantly higher mean counts of anaerobic species, such as *T. forsythia*, *P. gingivalis*, and *T. denticola*, in the supragingival environment of diseased implants compared with healthy implants. These are referred to as indicator microorganisms or key periodontal pathogens.

In recognizing the importance of implant maintenance programs in preventing peri-implantitis by minimizing bacterial colonization, there is a critical need to develop implant modifications that mitigate bacterial colonization, proliferation, and surface corrosion [5]. Bacterial adhesion and colonization are complex processes influenced by factors such as the environment, bacterial characteristics, and material surface properties, including chemical composition and topography [6].

Anodized titanium nanotube surfaces are nanostructures produced by surface modification and have emerged as a promising strategy for surface modification with the potential to exhibit antibacterial properties [1,5]. They are easy to fabricate and exhibit good biocompatibility [1]. Studies [7,8] have demonstrated reduced bacterial proliferation on titanium nanotube surfaces compared with implant surfaces without nanotube modifications. The complex antibacterial mechanisms primarily stem from the nanotube geometry and physicochemical properties, including charge repulsion, membrane stretching, and surface roughness variation [1].

The size of the nanotube is an important parameter that may affect the nanotube’s antibacterial properties. However, there has yet to be a consensus regarding the ideal nanotube diameter. Peng et al. [9] demonstrated that a larger diameter is associated with a higher antibacterial ability. Radtke et al. [10] found that smaller diameters exhibited a more significant antibacterial effect. These discrepancies are attributed to the inability to control variables precisely during the experiments [1].

Biocompatible thin film coatings on nanotube surfaces have been explored to enhance antibacterial capabilities. Various coatings, including silica-based coatings, have been tested to improve the antibacterial properties of dental implant surfaces [11]. For instance, Mokhtari et al. [12] demonstrated bactericidal solid abilities with a silica-based coating on titanium nanotube surfaces. Previous research on titanium discs revealed the potential of silicon carbide (SiC) to protect surfaces against bacterial corrosion after 30 days and minimize *P. gingivalis* proliferation after 4 h [3,11]. Coating 100 nm titanium nanotube surfaces with SiC showed comparable antibacterial effects against *P. gingivalis*, *T. forsythia*, and *T. denticola* compared with non-coated 100 nm titanium nanotubes [5].

Drawing upon the findings of previous research, the present study delves into the antibacterial effect of nanotube titanium surfaces against *P. gingivalis* and *T. denticola*. Specifically, our research seeks to investigate whether the nanotube diameter and/or the presence of a SiC coating influence bacterial proliferation on the surface. Based on the literature, we aimed to test the hypothesis that there is a significant difference in bacterial proliferation between titanium surfaces with: (1) 50 nm and 100 nm diameter nanotubes; (2) SiC-coated and non-coated nanotubes.

## 2. Materials and Methods

### 2.1. Experimental Design

A laboratory-based investigation was conducted utilizing 72 nanotubular anodic titanium oxide films (InRedox, Longmont, CO, USA) with dimensions of 0.03 mm × 5 mm × 5 mm. These titanium nanotube sheets were fabricated using anodization titanium oxide, resulting in nanotube diameters of 50 ± 10 nm and 100 ± 20 nm.

Different-sized nanotubes were characterized previously and reported in a different study [13]. The nanotubes and SiC-coated nanotubes were examined using an energy-dispersive X-ray spectroscopy (EDS) analysis to determine the composition of the surface and showed the main elements as Ti, O, F, and Al from the non-coated nanotubes and additional Si elements on the coated nanotubes. The results were consistent between all the samples with different nanotube diameters. Electron microscopy (TEM) was used to determine the coating morphology on the internal surface of the nanotubes, and it was demonstrated that the coating covered the internal surface of the nanotubes entirely.

The study comprised four distinct groups: (1) non-coated 50 nm titanium nanotubes, (2) silicon carbide (SiC)-coated 50 nm titanium nanotubes, (3) non-coated 100 nm titanium nanotubes, and (4) SiC-coated 100 nm titanium nanotubes. Each group had a total of 18 samples.

### 2.2. Coating Process

Thirty-six samples (18 with 50 nm and 18 with 100 nm nanotube diameter) were coated with SiC using a plasma-enhanced chemical vapor deposition (PECVD, PlasmaTherm 790 system, St. Petersburg, FL, USA) technique. Before the deposition, meticulous surface preparation procedures were executed. This involved thorough cleaning of the samples using acetone followed by isopropyl alcohol. Subsequently, the samples were dried using compressed nitrogen and treated with ozone to eliminate surface carbon contamination.

During the PECVD process, silicon dioxide (SiO_2_) and silicon carbide (SiC) dielectric films were applied to the titanium nanotube sheets. The deposition conditions were precisely calibrated, with 2 nm SiO_2_ deposited before 8 nm SiC. The temperature was maintained at 300 °C throughout the process, with a deposition rate of 330 Å/min for SiO_2_ and 170 Å/min for SiC. The precursors used for the SiO_2_ film deposition included 5% silane (SiH_4_) balanced in helium and nitrous oxide (N_2_O), while methane (CH_4_) and silane (SiH_4_) served as precursors for the SiC film. A monolayer of nitrogen atoms was introduced onto the SiC surface by applying 5% ammonia (NH_3_) to the samples in the PECVD for 2 min. Following the deposition, the samples underwent thermal annealing. The coating thickness was 10 nm, with the sequential application of the SiO_2_ and SiC layers contributing to the desired coating characteristics.

### 2.3. Bacterial Proliferation

All the titanium sheets were sterilized in an autoclave (121 °C, 60 min) and were distributed on a sterile 24-well plate. This study used two predominant peri-implantitis bacteria, *P. gingivalis* (FDC 381) and *T. denticola* (ATCC 35405), to induce a monobacterial infection. *P. gingivalis* (FDC 381) was grown in a Brucella blood agar plate supplemented with hemin and vitamin K (Hardy Diagnostics, Santa Maria, CA, USA). The oral spirochete *T. denticola* (ATCC 35405) was grown in GM-1 broth. The bacteria were grown and maintained in a Coy anaerobic chamber at 37 °C for 3 days, as previously described [14,15].

*P. gingivalis* was harvested from the media plate using a sterile cotton tip applicator. The log-phase culture of *T. denticola* was harvested using centrifugation (8000 rpm for 10 min), and the pellet was washed once with phosphate-buffered saline (PBS). *P. gingivalis* was suspended in Brucella broth, and *T. dentcola* was suspended in GM-1 broth and vortexed vigorously. The axenic nature of the bacteria was assessed using Gram staining. The number of bacterial cells for the infection was determined using the Petroff–Hausser bacterial counting chamber. The bacteria were diluted in respective media broths to reach the final concentrations of 10^10^ cells/mL for *P. gingivalis* and 2 × 10^8^ cells/mL for *T. denticola*.

The samples were taken in triplicate, and 1 mL of bacterial suspension was added to each well containing a nanotube sheet. The samples were cultivated for 3 and 7 days, with fresh media replenishments every 3 days. The control samples for each group were maintained without bacteria but with the same culture media for 7 days, with media replenishments every 3 days.

In addition to Gram staining, bacterial-specific 16S rRNA gene-specific primers that amplify specific 16S rRNA genes using the Phusion High Fidelity Master Mix (New England Biolabs (NEB), Ipswich, MA, USA) were performed as described previously [14,15]. After removing the bacterial suspension from each well, a colony polymerase chain reaction (PCR) test was performed using a Bio-Rad Thermal Cycler, Hercules, CA, USA, with the *P. gingivalis*-specific 16S rRNA gene-specific forward primer 5′-GGT AAG TCA GCG GTG AAA CC-3′, the reverse primer 5′-ACG TCA TCC ACA CCT TCC TC-3′, the *T. denticola*-specific 16S rRNA gene-specific forward primer 5′-TAA TAC CGA ATG TGC TCA TTT ACAT-3′, and the reverse primer 5′-CTG CCA TAT CTC TAT GTC ATT GCT CTT-3′. The DNA of the respective bacteria was used as a template for a positive control, and the absence of any bacterial DNA was used as a negative control. The PCR products were run on 1% agarose gel electrophoresis and visualized under the UVP GelStudio touch Imaging System (Analytik Jena US LLC, Upland, CA, USA).

### 2.4. Fluorescence Assay for Bacteria

After 3 and 7 days of incubation, bacterial samples adhering to the nanotube surfaces were processed for analysis. Each experimental group’s bacterial specimens from two samples were carefully selected and subjected to fixation using a 3.7% formaldehyde solution for 15 min. Following fixation, the specimens were stained utilizing the SYTO^®^ 9 dye from the Live/Dead BacLight Bacterial Viability Kit, sourced from ThermoFisher Scientific, headquartered in Waltham, MA, USA. The staining process was carried out over 30 min to ensure optimal penetration and staining of the bacterial cells.

Subsequently, the stained bacterial specimens were examined using a Zeiss Imager A2 microscope (Carl Zeiss AG, Oberkochen, Germany). Utilizing this microscopy system, fluorescence images of the live and dead bacteria were captured. The imaging process was conducted at a magnification level of 10×, allowing for the visualization and analysis of the bacterial distribution to the nanotube surfaces. In addition, the ImageJ software version 1.38e analyzed two random fluorescence images of each group to calculate the bacteria coverage percentages.

### 2.5. Scanning Electron Microscopy

Following the incubation periods of 3 and 7 days, the assessment of the bacterial adhesion on the nanotube surfaces was conducted using scanning electron microscopy (SEM). For this purpose, one sample from each experimental group was prepared for the SEM analysis using a standardized protocol.

Firstly, the bacteria adhering to the samples were fixed using a fixative solution of 3% glutaraldehyde, 0.1 mol/L sodium cacodylate, and 0.1 mol/L sucrose. The fixation process was carried out for 45 min to ensure the optimal preservation of the bacterial specimens for the subsequent analysis.

Following fixation, the samples underwent a series of processing steps to prepare them for the SEM imaging. This included immersion in a buffer solution containing 0.1 mol/L sucrose and 0.1 mol/L sodium cacodylate for 10 min to ensure proper stabilization of the specimens. Subsequently, the samples were subjected to serial ethanol dehydration, with each dehydration step lasting 10 min, to remove any residual moisture and prepare the specimens for further processing.

Following dehydration, the samples were further treated with hexamethyldisilazane (HDMS) to facilitate efficient drying and preservation of the sample morphology. Once thoroughly dehydrated, specimens were subjected to sputter-coating with a palladium–gold alloy using the Polaron SC 7620 Sputter Coater (Quorum Technologies, Lewes, UK). This coating process, with a thickness of 10 nm, was essential for reducing the charging effects during the SEM analysis, ensuring accurate imaging and analysis of the bacterial specimens.

Finally, SEM imaging was performed utilizing the FEI NOVA NanoSEM 430 system (FEI Company, Hillsboro, OR, USA). Operating at 10 kV with a spot size of 3.5 µm under high vacuum conditions, the SEM system provided high-resolution imaging to analyze the bacterial adhesion on the nanotube surfaces. Images were captured at a magnification of 2000×, allowing for comprehensive visualization and analysis of the bacterial distribution on the nanotube surfaces.

## 3. Results

**Figure 1:**
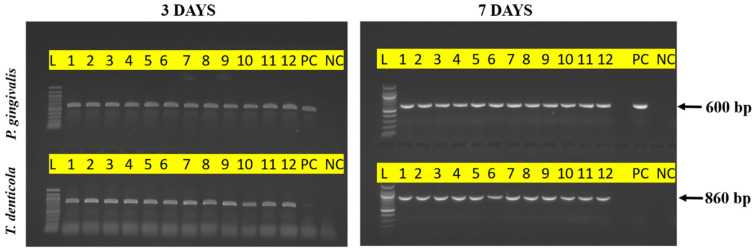
PCR analysis of titanium nanotube samples with a monobacterial infection (*P. gingivalis* or *T. denticola*). Agarose gel showing the 16S rRNA gene amplified from the respective supernatants samples taken after 3 and 7 days of incubation with *P. gingivalis* and *T. denticola*. All the samples demonstrated the presence of 16S rRNA gene amplicons of the respective bacteria. L: 100 bp DNA Ladder; 1 to 3: non-coated 50 nm samples; 4 to 6: SiC-coated 50 nm samples; 7 to 9: non-coated 100 nm samples; 10 to 12: SiC-coated 100 nm samples; PC-Positive Control; NC-Negative Control.

16S rRNA gene amplification from each sample showed the presence of the respective bacterial 16S rRNA gene amplicons. After monobacterial infection, both *P. gingivalis* and *T. denticola* were detected in all the samples of non-coated 50 nm titanium nanotubes, SiC-coated 50 nm titanium nanotubes, non-coated 100 nm titanium nanotubes, and SiC-coated 100 nm titanium nanotubes samples at 3 and 7 days (Figure 1). These results indicated that both pathogens could colonize the nanotube samples, regardless of the nanotube diameter or the presence of the SiC coating, and other bacterial contamination was not observed during the experiment.

**Figure 2:**
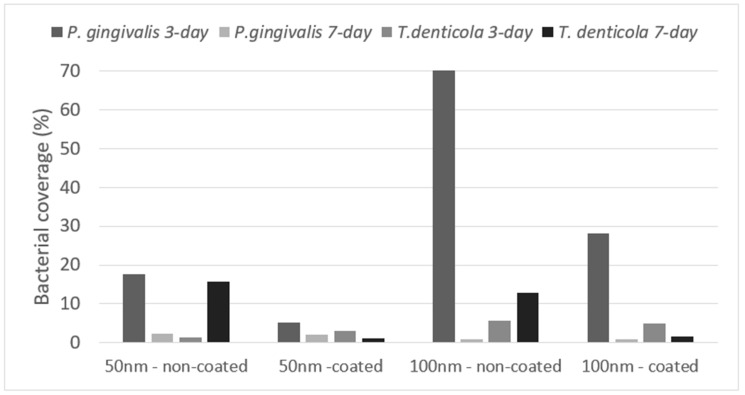
Bacterial coverage on the titanium nanotube surfaces coated and non-coated with silicon carbide.

The results for the bacterial coverage (Figure 2) showed coverage lower than 20% for all the groups, except for the 100 nm coated and non-coated samples incubated with *P. gingivalis*. Bacterial coverage was reduced from 3 to 7 days for all the groups except for the 50 and 100 nm non-coated samples incubated with *T. denticola*. For the other groups, the bacterial coverage was smaller than 2.5% at the 7-day time point.

**Figure 3:**
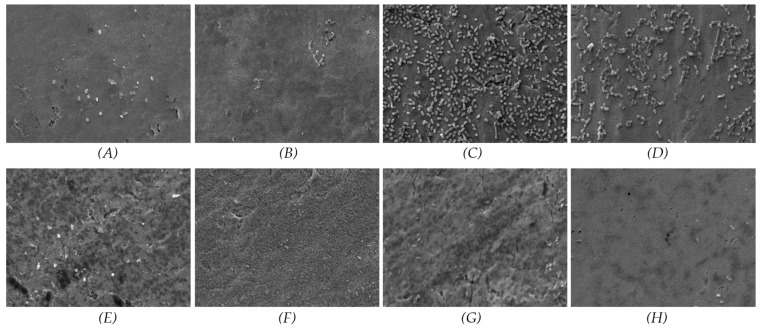
SEM images of *P. gingivalis* cultured for 3 and 7 days on coated and non-coated titanium nanotube samples. (**A**) 50 nm non-coated at 3 days; (**B**) 50 nm coated at 3-day; (**C**) 100 nm non-coated at 3 days; (**D**) 100 nm coated at 3 days; (**E**) 50 nm non-coated at 7 days; (**F**) 50 nm coated at 7 days; (**G**) 100 nm non-coated at 7 days; (**H**) 100 nm coated at 7 days.

**Figure 4:**
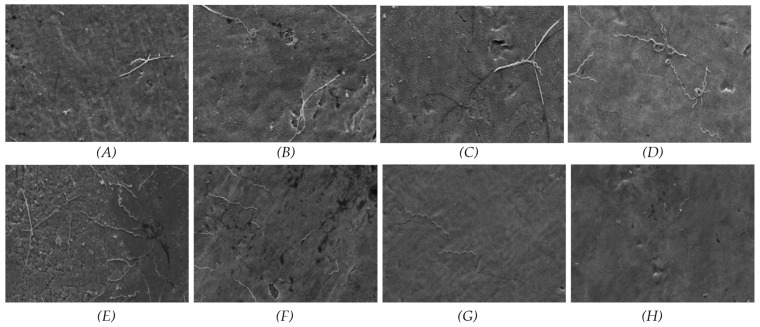
SEM images of *T. denticola* cultured for 3 and 7 days on coated and non-coated titanium nanotube samples. (**A**) 50 nm non-coated at 3 days; (**B**) 50 nm coated at 3 days; (**C**) 100 nm non-coated at 3 days; (**D**) 100 nm coated at 3 days; (**E**) 50 nm non-coated at 7 days; (**F**) 50 nm coated at 7 days; (**G**) 100 nm non-coated at 7 days; (**H**) 100 nm coated at 7 days.

The results for *P. gingivalis* at the 3-day time point indicated that the SiC coating effectively prevented bacterial adherence on both the 50 nm and 100 nm samples. Furthermore, a substantial decrease in bacterial coverage was observed for the 100 nm samples from day 3 to day 7. In contrast, the results for *T. denticola* at 3 days showed low bacterial coverage across all groups. However, in the non-coated groups, bacterial coverage was increased from day 3 to day 7, suggesting that the SiC coating exerted an antibacterial effect. Figure 3 and Figure 4 present the SEM images of the samples inoculated with *P. gingivalis* and *T. denticola.*

**Figure 5:**
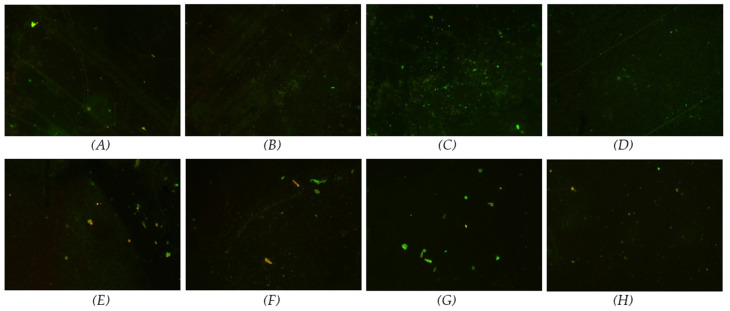
Live/dead fluorescence images of *P. gingivalis* cultured for 3 and 7 days on coated and non-coated titanium nanotube samples (stain marks live bacteria in green and dead in red). (**A**) 50 nm non-coated at 3 days; (**B**) 50 nm coated at 3 days; (**C**) 100 nm non-coated at 3 days; (**D**) 100 nm coated at 3 days; (**E**) 50 nm non-coated at 7 days; (**F**) 50 nm coated at 7 days; (**G**) 100 nm non-coated at 7 days; (**H**) 100 nm coated at 7 days.

**Figure 6:**
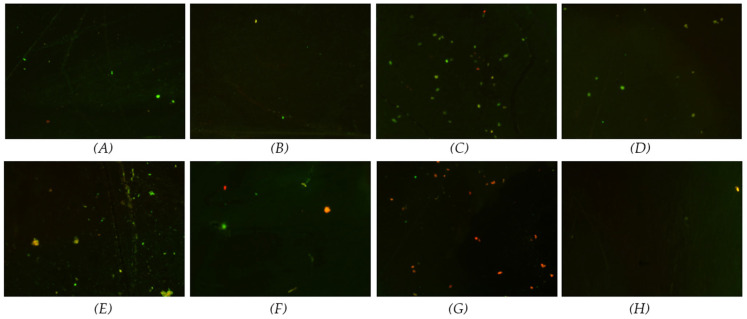
Live/dead fluorescence images of *T. denticola* cultured for 3 and 7 days on coated and non-coated titanium nanotube samples (stain marks live bacteria in green and dead in red). (**A**) 50 nm non-coated at 3 days; (**B**) 50 nm coated at 3 days; (**C**) 100 nm non-coated at 3 days; (**D**) 100 nm coated at 3 days; (**E**) 50 nm non-coated at 7 days; (**F**) 50 nm coated at 7 days; (**G**) 100 nm non-coated at 7 days; (**H**) 100 nm coated at 7 days.

Figure 5 and Figure 6 display the fluorescence images stained with SYTO^®^ 9, which marks the live bacteria in green and the dead bacteria in red. The fluorescence images revealed that most bacteria were alive at the 3-day time point. By the 7-day time point, a slight increase in dead *P. gingivalis* was observed compared with the 3-day time point, although most bacteria remained alive (Figure 5). In contrast, for *T. denticola*, there was a notable increase in the number of dead bacteria at the 7-day time point, particularly in the coated samples, where the dead bacteria outnumbered the live bacteria (Figure 6).

## 4. Discussion

Dental implants are widely recognized for their exceptional long-term success rates and represent a pivotal treatment modality for addressing tooth loss. Despite their overall effectiveness, instances of implant failure persist, emphasizing the critical importance of identifying the associated risk factors [16]. Peri-implant diseases, notably peri-implantitis, are significant contributors to dental implant failure, often attributed to bacterial colonization by pathogens such as *P. gingivalis* and *T. denticola*. A crucial event initiating infection involves bacterial adherence to the implant surface, prompting extensive exploration into surfaces with inherent properties that inhibit bacterial attachment as a potential strategy for reducing implant failure rates [17].

The present study delves into the comprehensive evaluation of a silicon carbide (SiC) coating applied to titanium nanotube surfaces, explicitly investigating the influence of the nanotube diameter on the inhibition of *P. gingivalis* and *T. denticola* proliferation. The results indicate that both the nanotube diameter and the application of the SiC coating exert influence on restricting bacterial proliferation on titanium surfaces.

Upon interaction with the human body, nanotube titanium surfaces demonstrate a remarkable characteristic of being biologically inert and safe [18]. This intrinsic property has positioned anodized titanium as a promising material for biomedical applications. Managing bacterial species on medical devices and implants represents a critical challenge. With the growing threat of antimicrobial resistance and the complexities associated with biofilm formation, the need to effectively control bacterial colonization has become increasingly urgent. Due to its unique surface characteristics, nanostructured titanium has garnered significant attention in biomedical research as a potential solution to address these challenges.

Researchers have extensively explored the antimicrobial potential of nanostructured titanium in various biomedical applications. Surface modification techniques have emerged as promising strategies for enhancing the properties of implants and medical devices [19]. By precisely manipulating the surface morphology and chemistry at the nanoscale, researchers aim to design surfaces that resist bacterial adhesion and actively inhibit bacterial proliferation.

The multifaceted antibacterial mechanisms exhibited by nanotube titanium surfaces involve intricate processes, such as charge repulsion, membrane stretching, and surface roughness. These mechanisms collectively contribute to the surface’s ability to resist bacterial adhesion and proliferation, enhancing its antimicrobial properties. One of the primary mechanisms at play is charge repulsion, which occurs due to the negative charges present on the titanium nanotubes and bacterial surfaces. This electrostatic repulsion impedes the initial adhesion of the bacteria to the nanotube surface, acting as a deterrent against bacterial colonization [20]. Furthermore, membrane stretching induced by the unique topography of the titanium nanotubes plays a crucial role in accelerating bacterial death. As the bacteria come into contact with the nanotubes, mechanical forces are exerted on the bacterial membrane, leading to membrane deformation and eventual rupture. This process, known as membrane stretching, effectively incapacitates the bacteria, rendering them unable to grow and causing their eventual demise [21,22].

In addition, these antibacterial mechanisms are intricately linked to the dimensions of the titanium nanotubes; however, there is no consensus on the relationship between the diameter of a titanium nanotube and its antibacterial properties. Divergent findings persist across studies, adding complexity to the understanding of this association. Noteworthy, Peng et al. [9], studying *S. epidermidis* proliferation with 30 to 80 nm nanotubes, asserted that increased diameters correlated with enhanced hydrophilicity, thereby augmenting the antibacterial capabilities. However, the present results indicate better results for small diameters, i.e., 50 nm. Radtke et al. [10], studying *S. aureus* proliferation on nanotubes with 15 to 80 nm, also found that the smaller-diameter titanium nanotubes exhibit a more robust antibacterial effect than their counterparts with larger diameters. The different methods and microorganisms can explain these divergences.

The present study showed different results for *P. gingivalis* and *T. denticola* with the non-coated samples. In contrast, the bacterial coverage decreased from 3 to 7 days for *P. gingivalis*, and the opposite occurred for *T. denticola.* The initial bacterial attraction to a material surface involves various forces, such as van der Waals or gravitational forces. Subsequent bacterial adhesion is reinforced by pili, leading to colony formation and the secretion of a biofilm layer rich in polysaccharides and proteins, protecting the immune system. Each species has unique surface structures, such as fimbriae, pili, and lipopolysaccharides, which interact differently with the surface. *P. gingivalis* fimbriae, for instance, are known to facilitate strong adhesion to various surfaces [23].

The ability to form biofilms can also differ significantly. *P. gingivalis* is a known primary colonizer with robust biofilm-forming capabilities, whereas *T. denticola* is often associated with established biofilms [23]. Their roles in biofilm development and stability can lead to different adhesion and proliferation patterns. Surface properties, such as roughness, hydrophobicity/hydrophilicity, and surface energy, can also play a role in this result.

Silicon carbide (SiC) coatings on titanium surfaces represent a significant area of interest in biomedical research, aiming to enhance the antibacterial properties of titanium implants and medical devices. Researchers, such as Bhaskar et al. [24], have conducted studies investigating the efficacy of SiC-based coatings on commercial pure titanium, particularly in comparison to other coatings such as diamond-like carbon (DLC). Their findings revealed promising results, indicating the effective inhibition of bacterial proliferation on SiC-coated surfaces, thus confirming the bactericidal effect of SiC coatings.

Despite the evident antibacterial efficacy of SiC coatings found in the present study, the precise mechanisms underlying their bactericidal properties remain somewhat elusive. However, researchers have proposed several hypotheses to elucidate these mechanisms. One such hypothesis revolves around hydrophilic functional groups within SiC coatings. These functional groups create electrostatic repulsion forces when the bacteria come into contact with the coated surface. The electrostatic repulsion impedes bacterial adhesion and colonization, inhibiting bacterial proliferation and biofilm formation [25].

Furthermore, the unfavorable surface energy of SiC-coated surfaces for bacterial adhesion is believed to contribute significantly to their antibacterial properties. The surface energy characteristics of SiC coatings render them less conducive to bacterial attachment than untreated titanium surfaces or surfaces coated with alternative materials. This unfavorable surface energy creates a hostile environment for bacterial colonization, further enhancing the bactericidal effect of SiC coatings [25].

The study by Ching et al. [26] sheds light on the remarkable antibacterial properties of silicon carbide (SiC) coatings, particularly when combined with a nitrogen monolayer on the SiC surface. The researchers observed a significant decline in bacterial activity, specifically a reduction of over 80% in *S. mutans* colonies when the SiC coating was layered with a nitrogen monolayer. One of the critical insights from their findings is the pivotal role played by the nitrogen monolayer in enhancing the antibacterial efficacy of the SiC coatings. This enhancement is attributed to the unique properties imparted by the nitrogen monolayer, particularly its ability to introduce a positive charge to the surface. Introducing a positive charge on the SiC-coated surface alters the electrostatic interactions between the surface and bacterial cells. This alteration disrupts the integrity of the bacterial cell membrane, leading to structural damage and dysfunction. As a result, bacterial cells experience increased permeability and eventual lysis, leading to cell apoptosis and death.

This investigation represents a crucial step towards elucidating the intricate mechanisms underlying bacterial adhesion and proliferation on dental implant surfaces. By elucidating the role of the nanotube diameter and surface coating in modulating bacterial behavior, our study contributes valuable knowledge to the ongoing quest for developing innovative strategies to enhance dental implant materials’ biocompatibility and antimicrobial properties. Ultimately, our findings hold immense potential for informing the design and development of next-generation dental implants with improved resistance to peri-implant infections, thereby advancing dental implant therapy’s long-term success and durability. However, further long-term in vitro investigations are necessary to assess the potential extended antibacterial effects of the coating. Additionally, in vivo animal models and in situ trials are needed to explore the implications of these results for clinical application.

## 5. Conclusions

The differences observed in bacterial proliferation between the experimental groups indicates that the nanotube diameter and surface coating can modulate bacterial adhesion and proliferation. Specifically, small nanotube diameters and the presence of the SiC coating appear to impart an antibacterial effect.

## Data Availability

The data that support the findings of this study are available from the corresponding author upon reasonable request.
